# Intravascular Lithotripsy for Peripheral Arterial Disease: Outcomes From a Single-Center Experience

**DOI:** 10.7759/cureus.103548

**Published:** 2026-02-13

**Authors:** Kamran Hamid, NajuA’Isha Ibrahim, Amro Elboushi, Haren Wijesinghe, Mohammed Elsabbagh, Arul Ganeshan

**Affiliations:** 1 Vascular Surgery, University Hospitals Birmingham NHS Foundation Trust, Birmingham, GBR; 2 Interventional Radiology, University Hospitals Birmingham NHS Foundation Trust, Birmingham, GBR

**Keywords:** critical limb-threatening ischemia, endovascular stenting, intravascular lithotripsy, peripheral arterial disease, shockwave

## Abstract

Background

Intravascular lithotripsy (IVL) is increasingly used to treat heavily calcified lower-limb arterial disease. We audited outcomes from a UK vascular center to assess the safety and effectiveness of this modality.

Methods

This was a single-center prospective audit of consecutive patients undergoing IVL at a UK vascular center between March 2023 and September 2025. Demographic data, lesion characteristics (including peripheral artery calcification scoring system {PACSS} grade), procedural details, and outcomes were recorded. Primary outcomes assessed safety, measured by 30-day survival, 30-day amputation-free survival (AFS), and procedure-related complications (perforation, dissection, and distal embolization), and effectiveness, defined as target vessel recanalization. Secondary outcomes included 30-day hospital readmission and one-year AFS. Statistical analyses included Fisher’s exact test and t-tests, as appropriate, with Kaplan-Meier survival analysis. Follow-up duration ranged from two to 24 months.

Results

Forty-five patients were included, of whom 37 (82.2%) were men, with a median age of 76.0 years. Twenty-four patients (53.3%) were elective admissions. Most procedures involved the superficial femoral artery (SFA), above-knee popliteal, and tibial segments, with Rutherford classes predominantly 4-5. Technical success was achieved in 91% (n = 41) of cases, and immediate procedural complications were infrequent. The 30-day readmission rate was 24.2% (n = 11). Severe calcification (PACSS grades 4-5) was not associated with major amputation (p = 0.67) or peri-procedural complications (p = 1.00). Thirty-day AFS was 75.6% (n = 37; 95% CI: 69.4%-91.7%), and 30-day overall survival was 88.9% (n = 40). Thirty-day mortality was significantly higher following emergency admission compared to elective admission (p = 0.03). At one year, major amputation occurred in 21.4% (n = 9) and all-cause mortality in 9.5%. Thirty-day readmission strongly predicted subsequent major amputation (66.7% versus 15.6%; p = 0.006).

Conclusions

In a predominantly critical limb-threatening ischemia (CLTI) cohort with heavy arterial calcification, adjunctive IVL achieved high technical success with low peri-procedural complication rates. Early limb and survival outcomes were largely driven by baseline disease severity and the urgency of presentation, with emergency admission and early readmission serving as adverse prognostic markers. IVL appears to be a safe and effective vessel preparation strategy in complex calcified disease, supporting the need for larger prospective studies to refine patient selection and long-term outcomes.

## Introduction

Peripheral arterial disease (PAD) is caused by the buildup of lipid deposits within the tunica media and intima of arteries; in the lower limbs, this results in restricted blood flow [1]. PAD frequently coexists with moderate-to-severe arterial calcification, which is particularly common in patients with diabetes and those on dialysis with end-stage renal disease [2]. Chronic limb ischemia represents the symptomatic manifestation of PAD and may eventually progress to a critical form, characterized by tissue loss or gangrene, nonhealing ulcers, and/or rest pain [3-8].

The Rutherford classification system is widely used to categorize chronic limb ischemia, encompassing a spectrum ranging from asymptomatic ischemia to extensive gangrene involving the lower limb.

For most clinical purposes, the ankle-brachial pressure index (ABPI), in conjunction with clinical findings, is used to identify and stratify critical limb-threatening ischemia (CLTI). A normal ABPI ranges from 1.0 to 1.4. Values below 0.9 are considered diagnostic of PAD, while values less than 0.5 are consistent with CLTI [3-8].

Revascularization strategies in such patients include either open surgical procedures or, in suitable candidates, endovascular therapies. Endovascular approaches primarily involve percutaneous transluminal angioplasty (PTA) using plain balloon angioplasty (PBA), drug-coated balloons (DCB), or intravascular lithotripsy (IVL) to disrupt calcification and prepare vessels for PBA, stent deployment, or DCB use [9,10].

For over 30 years, lithotripsy has been used in upper gastrointestinal and urological surgery to facilitate the breakdown of calcium-containing lesions in the gallbladder and renal tract, respectively. The application of shockwave lithotripsy to the cardiovascular system was first explored in 2007 by Daniel Hawkins, a businessman, and John Adams, an electrical engineer. This technology was initially applied to coronary artery disease, where systematic reviews and meta-analyses demonstrated its safety and effectiveness [11]. The extrapolation of IVL technology from the coronary to the peripheral circulation was a pragmatic step to achieve similar outcomes in heavily calcified peripheral arterial disease. The IVL device consists of a 0.014-inch guidewire with an array of lithotripsy emitters enclosed within an integrated balloon. Once the balloon is positioned at the site of calcific lesions within lower-limb vessels, the emission of sonic pressure waves fractures calcium located in the intimal and medial layers of the vessel wall. Consequently, arteries that were previously inaccessible or associated with high recanalization failure rates can be treated more effectively [12].

The presence of arterial calcification increases the risk of elastic recoil, dissection, and failure to achieve adequate vessel patency. Severely calcified iliac arteries may also preclude the navigation of larger delivery systems required for supra-inguinal endovascular interventions, such as endovascular aortic aneurysm repair (EVAR). This further complicates endovascular therapy by limiting vessel expansion and increasing the risk of dissection, perforation, and recoil [11,12].

The severity of lesion calcification can be assessed using the peripheral artery calcification scoring system (PACSS), which ranges from grade 0 (no visible calcium at the target lesion site) to grade 4 (bilateral calcification extending ≥5 cm) [11,12].

Meta-analyses and pooled data have demonstrated improved procedural success with IVL compared to standard angioplasty in calcified femoropopliteal lesions, with low peri-procedural complication rates. Against this backdrop, we evaluated IVL outcomes at our center [12-14].

## Materials and methods

Design and setting

A single-center prospective audit was conducted to collect data on patients undergoing angioplasty involving intravascular lithotripsy (IVL) technology at a major vascular surgery center in the United Kingdom, following approval from the Novel Therapeutics Committee (NTC) of the University Hospitals Birmingham NHS Foundation Trust (approval number: NT009/22). After every 10 patients, the NTC was updated on procedural outcomes to ensure the ongoing safety of this novel technique.

Participants

Forty-five patients with either critical limb-threatening ischemia (CLTI; Rutherford classes 4-6) or peripheral arterial disease requiring adjunctive access intervention to facilitate endovascular aortic procedures were included. Patients were selected if significant calcification was identified in the target arterial lesion on computed tomography angiography (CTA) or intraoperatively during angiography; these patients were therefore offered IVL and included in the study. Selection was based on operator choice (consultant vascular surgeon or interventional radiologist). Diseased arterial segments ranging from the iliac arteries to the infra-popliteal arteries were eligible for inclusion. The device used was the peripheral IVL system (Shockwave Medical Inc., Santa Clara, CA).

Data collection

Informed consent was obtained from all patients included in this audit. Patient sex was reported according to biological sex at birth.

Collected variables included demographics, comorbidities, the mode of admission, lesion characteristics, procedural details, procedural outcomes, and Rutherford classification.

Outcome measures

The primary outcomes were the safety and effectiveness of IVL. Safety was assessed by 30-day mortality, 30-day amputation-free survival (AFS), and procedure-related complications, including arterial perforation, dissection, and distal embolization. Effectiveness was evaluated by successful target vessel recanalization.

Secondary outcomes included 30-day hospital readmission and one-year amputation-free survival.

Statistical analysis

Continuous variables were presented as mean ± standard deviation (SD) or median with interquartile range (IQR), as appropriate, while categorical variables were reported as counts and percentages. Kaplan-Meier survival curves were generated for 30-day overall survival, 30-day amputation-free survival, and one-year amputation-free survival. Statistical analyses were performed using Python (Python Software Foundation, Wilmington, DE).

## Results

Forty-five patients were included in the study, of whom 37 (82.2%) were men. The median age was 76.0 years (IQR: 64.0-81.0), with a mean age of 73.4 ± 10.6 years. Most patients had diabetes mellitus and hypertension. Comorbidities and their frequencies are detailed in Table 1.

**Table 1:** The list of patients’ comorbidities. COPD: chronic obstructive pulmonary disease

Comorbidities	N (%)
Diabetes mellitus	31 (68.9%)
Hypertension	20 (44.4%)
Ischemic heart disease	13 (28.9%)
Chronic kidney disease	8 (17.8%)
Stroke	6 (13.3%)
Atrial fibrillation	5 (11.1%)
COPD	5 (11.1%)
Congestive cardiac failure	4 (8.9%)
Hepatocellular carcinoma	2 (4.4%)
Prostate cancer	2 (4.4%)

Twenty-four patients (53.3%) underwent intervention electively, while 21 (46.6%) were admitted on an emergency or urgent basis. The median hospital length of stay was one day (IQR: 1-8 days), reflecting that many procedures were performed with short admissions (including day-case procedures), although a small number of patients required prolonged hospitalization of up to 38 days.

Lesion and procedural characteristics

**Figure 1:**
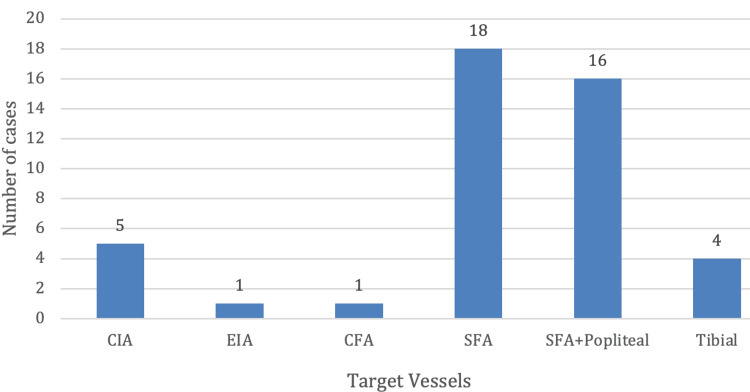
Anatomic distribution of the segments treated with IVL. CIA, common iliac artery; EIA, external iliac artery; CFA, common femoral artery; SFA, superficial femoral artery; IVL, intravascular lithotripsy

Most treated lesions were located in the superficial femoral artery (SFA) and the above-knee popliteal artery. Many patients underwent treatment of multiple arterial segments during the same procedure (Figure 1). In four patients, the lesion could not be crossed or recanalized with a guidewire, and the procedure was therefore abandoned, resulting in a technical success rate of 91.1%.

The most common Rutherford classification was class 6, followed by class 4 and class 3. Four IVL procedures were performed to facilitate device delivery during endovascular aortic aneurysm repair (EVAR) at Rutherford class 2 (Table 2).

**Table 2:** The percentage of Rutherford classes treated by IVL. Others: IVL used for device delivery in the iliac vessels for endovascular aortic aneurysm repair (EVAR). IVL: intravascular lithotripsy

Rutherford class	Number	Percent of total
Class 4	1	2.2%
Class 5	6	13.3%
Class 6	34	75.5%
Class 2 (EVAR)	4	8.8%
Total	45	100%

The peripheral artery calcification scoring system (PACSS) grade (Table 3) was primarily calculated using computed tomography imaging reconstructed with dedicated software (TeraRecon Intuition Inc., Durham, NC) when available, supplemented by arterial duplex ultrasound where necessary. Severe arterial calcification (PACSS grades 4-5) was not significantly associated with amputation risk (p = 0.67) nor was the occurrence of peri-procedural complications (p = 1.00). Table 3 summarizes calcification severity.

**Table 3:** The distribution of the treated arterial segments according to the PACSS calcification grading system. PACSS: peripheral artery calcification scoring system

PACSS grade	Percentage
Grade 1	3 (6.6%)
Grade 2	6 (13.3%)
Grade 3	6 (13.3%)
Grade 4	19 (42.2%)
Grade 5	11 (24.4%)

Primary outcomes

**Figure 2:**
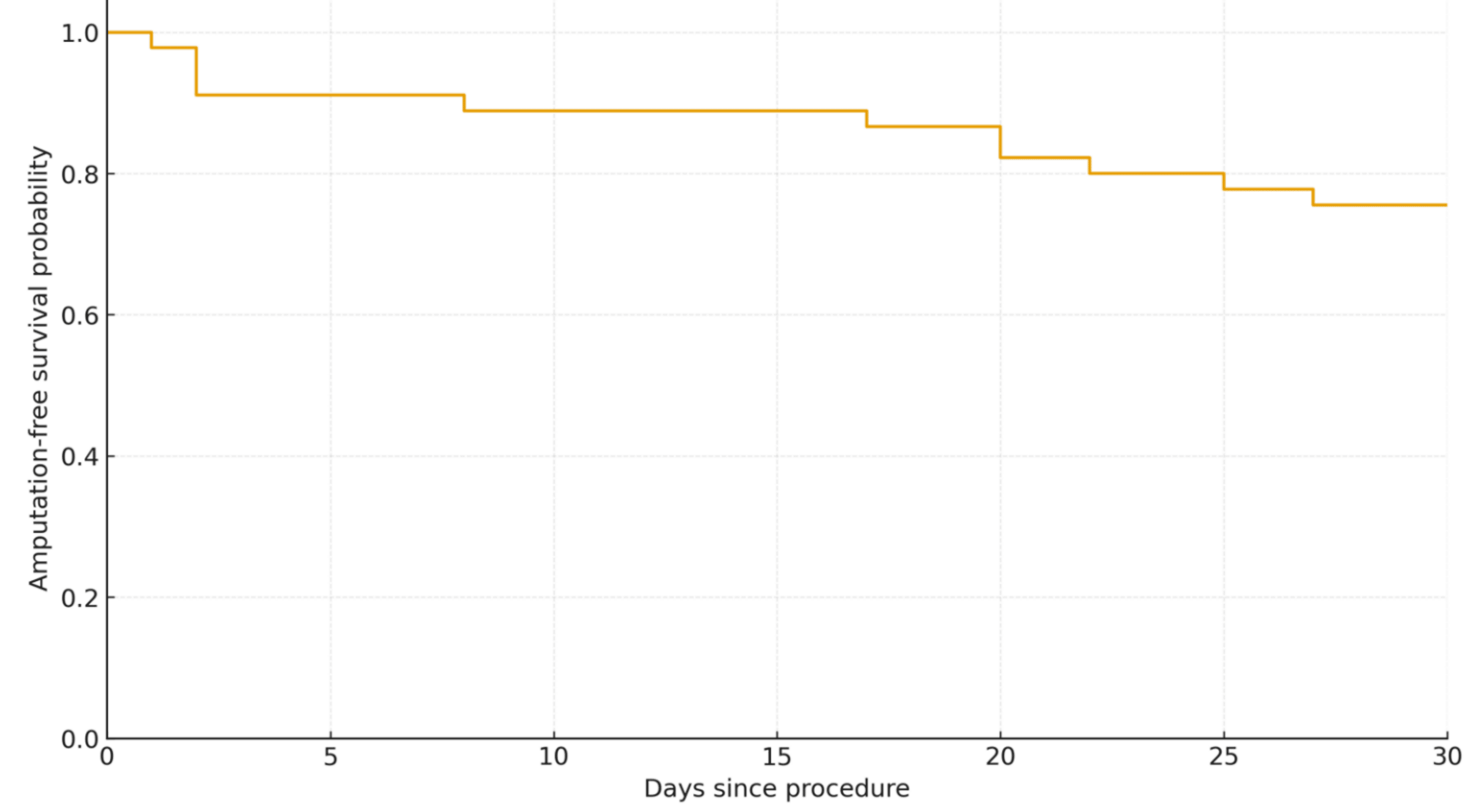
Kaplan-Meier curve of the 30-day AFS. AFS: amputation-free survival

Thirty-day overall amputation-free survival (AFS) was 75.6% (37/45; 95% CI: 69.4%-91.7%) (Figure 2). Thirty-day overall survival was 88.9% (40/45; 95% CI: 76.5%-95.2%) (Table 4 and Figure 2). Only one death was directly attributable to PAD presentation (CLTI); the remaining four deaths occurred in patients with significant multimorbidity and sepsis.

**Table 4:** The 30-day and one-year outcomes. AFS: amputation-free survival

Outcome	N (patients)	Events (within window)	Survival probability (95% CI)
30-day survival	45	5 deaths	88.8% (80.2%-98.6%)
30-day AFS (major amputation)	45	11 events (amputation or death)	75.6% (66.1%-95.8%)
1-year AFS (major amputation or death)	45	5 events (amputation or death)	66.7% (75.3%-95.2%)

**Figure 3:**
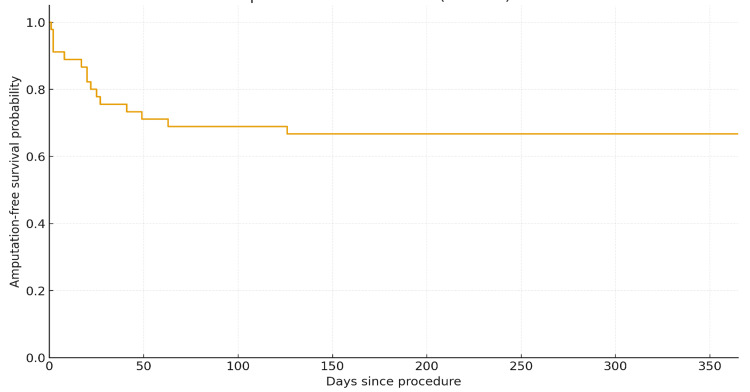
Kaplan-Meier curve of the one-year AFS. AFS: amputation-free survival

The Kaplan-Meier graphs showing AFS at one year suggest that after six months, the rate of amputation and deaths stabilized (Figure 3).

The patients who died were not significantly older than survivors (mean age of 74.0 versus 75.3 years, p = 0.61). There was no statistically significant difference by sex; one-year mortality was 8.6% in men and 16.7% in women (3/35 versus 1/6, p = 0.48), although the small sample size limits interpretation.

Procedural complications included arterial dissection (one patient), distal embolization (five patients), pseudoaneurysm formation (one patient), and failure to recanalize (four patients). None of these complications resulted in limb loss or mortality within 30 days (Table 5). The target vessel recanalization rate was 91% (41/45).

**Table 5:** Procedure complication up to 30 days. SFA, superficial femoral artery; EIA, external iliac artery

Type of complication	Count	Percent of 45	95% CI	Target vessels
Dissection	1	2.2	0.4%-12.1%	Infra-popliteal
Groin pseudoaneurysm	1	2.2	0.4%-12.1%	SFA
Distal embolization	5	11.1	3.7%-21.6%	SFA (four) and EIA (one)
Failure to recanalize	4	8.9	3.7%-21.6%	Infra-popliteal

Secondary outcomes

The 30-day hospital readmission rate was 24.2% (11/45; Wilson 95% CI: 16.5%-43.8%). Readmission was more frequent among emergency admissions compared to elective admissions, although this did not reach statistical significance (p = 0.070).

**Figure 4:**
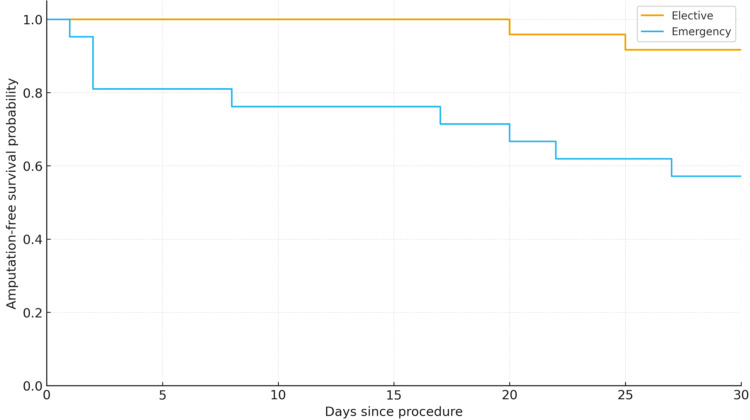
Thirty-day AFS according to the mode of admission. AFS: amputation-free survival

**Figure 5:**
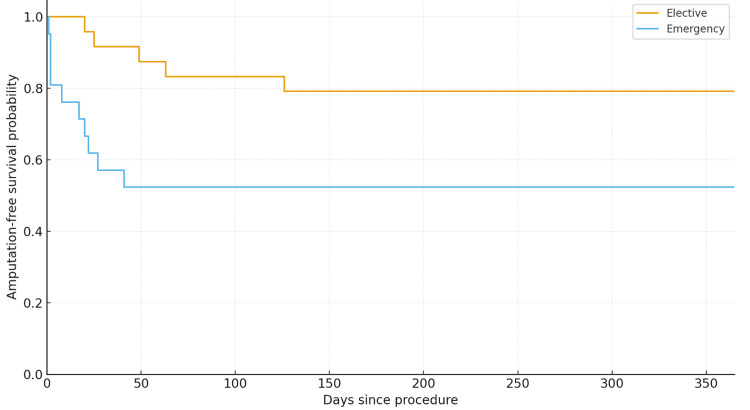
One-year AFS according to the mode of admission. AFS: amputation-free survival

Thirty-day mortality was significantly higher in patients admitted on an emergency basis compared to those admitted electively (p = 0.03) (Figure 4). This difference was also reflected in one-year AFS outcomes (Figure 5). The overall AFS at one year was 66% (Figure 3).

Non-elective admissions had a significantly higher one-year mortality rate compared to elective admissions (21% versus 0%, p = 0.04; Fisher’s exact test). In contrast, one-year major amputation rates were similar between non-elective and elective groups (approximately 21% in each, p = 1.00), indicating no significant association between admission urgency and amputation incidence.

The strongest association observed was between 30-day readmission and subsequent major amputation. The patients who ultimately required major amputation were significantly more likely to have been readmitted within 30 days of the index IVL procedure. Nearly two-thirds (66.7%) of the patients who underwent amputation were readmitted within 30 days, compared to only 15.6% of those who did not require amputation (p = 0.006; Fisher’s exact test).

Patients undergoing amputation were slightly older than those who did not (mean age: 77.1 versus 74.6 years), but this difference was not statistically significant (p = 0.47). There was no significant difference by sex; although none of the six female patients required major amputation (0% versus 25% of men), this trend did not reach statistical significance (p = 0.31; Fisher’s exact test).

## Discussion

The calcification of arteries results in significant difficulty during endovascular therapy for peripheral arterial disease. This includes difficulty in crossing diseased arterial segments with guidewires and the delivery of stents, reduced primary patency, and higher complication rates, such as dissection and vessel perforation. Intravascular lithotripsy (IVL) aims to address these challenges without increasing the risk profile of angioplasty.

IVL was used as an adjunctive treatment rather than as a primary modality of choice for all individuals in our cohort. This was associated with a high technical success rate of 91% (n = 40), which corresponds with other studies and meta-analyses conducted by Wong et al. [15,16]. Most studies and trials evaluating the safety and effectiveness of IVL did not include amputation-free survival (AFS) or all-cause mortality at 30 days or one year; instead, vessel patency was the primary outcome. In our single-center audit, the primary outcomes were 30-day and one-year AFS and overall survival.

DISRUPT PAD III, an industry-sponsored randomized trial, compared outcomes between IVL and plain balloon angioplasty (PBA). The trial cohort consisted predominantly of Rutherford class 3 patients (70%), with no patients in class 5 or 6. The trial demonstrated a technical success rate of 66.4% [10-12]. In contrast, our cohort consisted predominantly of patients with critical limb-threatening ischemia (CLTI; Rutherford classes 4-6), accounting for over 80% of cases. This was reflected by a high prevalence of rest pain and/or tissue loss as the presenting indication and a technical success rate of 91%. In the literature, Rutherford class 3 disease represents the most common severity (59%), with only approximately one-quarter of patients presenting with CLTI (Rutherford classes 4-6) [13-15].

We observed a 30-day major amputation rate of approximately 17% (8/45), which is higher than that reported in comparable studies (approximately 2.6%) [15-17]. Consequently, our estimated 30-day AFS was 75%, compared with Stavroulakis et al. [9] and Kohler et al. [18] at 98% and 58%, respectively, indicating that approximately one-third of the patients experienced early major limb loss or death [15-17]. Amputation-free survival at 12 months was approximately 66%, which is lower than the 89% reported by Stavroulakis et al. [9]. This lower AFS is attributable to the higher CLTI burden in our cohort.

Thirty-day all-cause mortality was approximately 11% (5/45). Specific data on all-cause mortality following IVL are limited in the literature, but reported rates are similar at around 11%. By one year, limb salvage and survival outcomes had stabilized: Freedom from major amputation was approximately 66%, and overall survival was approximately 85%-90%. These outcomes are comparable to those reported by Stavroulakis et al. [9] and Salihi et al. [17] (89% and 78%, respectively).

Our cohort included a greater proportion of severely ill patients with CLTI (nearly all with ischemic rest pain or ulcers), whereas Stavroulakis et al. included a mixed population of claudicants and patients with CLTI (only 56% CLTI) [9]. The lower-limb salvage observed in our series underscores the grave prognosis of advanced CLTI, even with modern endovascular therapy. By contrast, randomized trial data in less severe disease demonstrate minimal early adverse events. The DISRUPT PAD III trial (Tepe et al., 2021), which studied moderately calcified femoropopliteal lesions predominantly in claudicants, reported zero 30-day deaths or amputations. In that trial, IVL used as a vessel preparation strategy resulted in 30-day major adverse event rates of 0% (IVL) versus 1.3% (standard angioplasty), with no significant differences in clinical outcomes at 30 days. The stark contrast with our 30-day mortality (~11%) and amputation rate (~17%) highlights the more advanced disease state of our patients and aligns with known CLTI prognoses in real-world practice [12].

Notably, we identified early clinical factors associated with poorer outcomes. Patients requiring unplanned readmission within 30 days had significantly lower 30-day and one-year AFS compared to those without early complications (p < 0.01). Additionally, patients presenting emergently (urgent admissions for CLTI) demonstrated a trend toward worse limb salvage than those admitted electively.

Despite these outcomes, our results are consistent with the general safety profile of IVL reported in the literature. Madhavan et al. (2020) performed a pooled patient-level analysis of five IVL studies (336 patients), in which only approximately 25% had CLTI [10]. They demonstrated high procedural success and remarkably low complication rates, with only 1.2% of treated lesions experiencing flow-limiting complications such as perforation or embolization. Similarly, Stavroulakis et al. reported infrequent device-related complications (3% flow-limiting dissection, 1% perforation, and 1% distal embolization) [9]. In our audit, acute complication rates were comparably low. We observed no arterial perforations, abrupt vessel closures, or flow-limiting dissections requiring unplanned stenting. Five patients (11%) experienced peri-procedural distal embolization, all managed successfully with aspiration thrombectomy, and one patient developed a groin hematoma/pseudoaneurysm. These complication rates did not exceed expectations for interventions in heavily calcified vessels and did not translate into poorer limb salvage outcomes [9,18].

The treatment of the common femoral artery (CFA) with IVL is a novel approach, as standard practice remains common femoral endarterectomy (CFEA), a time-tested procedure. IVL use in the CFA has been reported by Stavroulakis et al. [19] and Del Canto Peruyera et al. [20], with cohorts of 33 and 15 patients, respectively. Both studies used drug-coated balloons adjunctively and reported procedural success rates of 97% and 100%, respectively. Distal embolization rates were 3%, and flow-limiting dissection occurred in 6%, requiring bailout stenting in 12% (n = 4) [19,20]. In our cohort, exclusive CFA IVL use was low at 2% (n = 1), as standard practice remains to offer CFEA. However, IVL may represent an appealing alternative in patients with CLTI and severe comorbidities, where open surgery under general anesthesia carries a high procedural risk [19,20].

The delivery of large endografts in diseased iliac arteries required the use of IVL in previously asymptomatic patients in 8% (n = 4) of cases, with a 100% technical success rate in our cohort. This compares favorably with previous studies by Fazzini et al. [21] and Price et al. [22], which reported 28 and nine cases, respectively, both achieving 100% technical success and each reporting one case requiring bailout stenting. Fazzini et al. also reported bilateral IVL use in eight cases, with an improvement of two Rutherford classes [21]. IVL use for transcatheter aortic valve implantation (TAVI)-related iliac vessel preparation has been reported in a meta-analysis by Sagris et al., demonstrating a success rate of 97%. In our study, the number of patients treated for this indication was small; these patients were previously asymptomatic, and none required bailout stenting [23].

Study limitations

This study represents a small case series with limited follow-up, which restricts the ability to achieve statistical significance for many variables.

## Conclusions

In our experience, IVL-facilitated angioplasty remained a safe and effective tool for the management of heavily calcified supra-inguinal and infra-inguinal arterial disease in high-risk patients with CLTI. Limb salvage achieved with IVL in this frail cohort, approximately 66% freedom from major amputation at one year, is notable, given that many patients were facing imminent amputation at presentation. In several cases, IVL-facilitated angioplasty was undertaken as a “last-chance” revascularization strategy in patients deemed poor candidates for bypass surgery or in whom standard balloon angioplasty had previously failed due to calcific recoil. IVL-facilitated angioplasty may therefore serve as an appealing endovascular alternative for patients with common femoral artery disease in whom open surgery carries a high anesthetic risk.
